# Prehabilitation before pancreatic surgery in the Netherlands: insights from a nationwide survey among pancreatic surgeons

**DOI:** 10.1186/s13741-025-00569-x

**Published:** 2025-07-31

**Authors:** Lis S. M. Hoeijmakers, Heleen Driessens, Carlijn I. Buis, Steven W. M. Olde Damink, Joost M. Klaase, Marcel den Dulk

**Affiliations:** 1 Department of Surgery, Institute of Nutrition and Translational Research in Metabolism (NUTRIM), Maastricht University, Maastricht, The Netherlands; 2 Division of Hepato-Pancreato-Biliary Surgery and Liver Transplantation, Department of Surgery, University Medical Center Groningen, Groningen, The Netherlands; 3 Department of Surgery, Maastricht University Medical Center+, Maastricht, The Netherlands; 4 Department of General, Visceral, Vascular and Transplant Surgery, University Hospital Essen, Essen, Germany

**Keywords:** Pancreatic surgery, Prehabilitation, Preoperative screening, Preoperative optimization

## Abstract

**Background:**

Prehabilitation programs are increasingly used to optimize patients before pancreatic surgery. A prehabilitation program should include screening, assessment, intervention, and reassessment of multiple patient-related modifiable risk factors. Consensus on the content of a prehabilitation program and which patients should receive prehabilitation is missing. This study aims to assess current preoperative screening practices, surgeons’ opinions, and knowledge of prehabilitation and identify existing prehabilitation programs for pancreatic surgery in the Netherlands.

**Methods:**

A nationwide descriptive cross-sectional study was conducted. All 15 hospitals providing pancreatic surgery in the Netherlands were included, and an online survey was sent to only one pancreatic surgeon per hospital. The survey was developed by the authors of this paper and based on a previously published survey for prehabilitation in colorectal surgery. Logical ordering and adaptive questioning were used.

**Results:**

All 15 surgeons responded, and they were all familiar with the term prehabilitation. Twelve hospitals (80%) offered prehabilitation, and in the majority of hospitals (7/12), prehabilitation was offered to all patients. Prehabilitation programs included multiple domains, whereby physical fitness and nutrition were most often included and mental resilience was the least often included domain. Each hospital implemented a different prehabilitation program in terms of included domains, screening methods, and interventions. For the majority of the domains, two or more different forms of screening and three or more different interventions were used across hospitals. A total of 53.3% of surgeons were willing to postpone the surgery of pancreatic malignancies up to a maximum of 4 weeks, 20% up to a maximum of 6 weeks, and 26.7% as long as necessary to optimize the patients’ preoperative overall fitness.

**Conclusions:**

Pancreatic surgeons in the Netherlands have knowledge of prehabilitation, but high variability exists in current practice regarding prehabilitation programs. There is a need for a uniform standardized prehabilitation program to be able to implement prehabilitation in the standard preoperative care pathway and enable comparison of results across hospitals.

**Supplementary Information:**

The online version contains supplementary material available at 10.1186/s13741-025-00569-x.

## Background

Prehabilitation is increasingly being recognized as a key strategy to optimize patients before elective abdominal surgery, including high-risk procedures such as pancreatic resection. Despite advancements in perioperative management, such as centralization of care, the introduction of enhanced recovery after surgery (ERAS) protocols, and mandatory auditing, surgery of the pancreas continues to be associated with a high risk of complications and postoperative morbidity (Jasmijn Smits et al. 2022; Büchler 2003). In addition, approximately one-fifth of patients who undergo pancreatic resection are considered frail (Wijk et al. 2021). The period between diagnosis and surgery presents a valuable window of opportunity for these patients to participate in a multimodal prehabilitation program to optimally prepare them for major surgery.

Multimodal prehabilitation has been shown to improve functional capacity and reduce postoperative complications in patients undergoing major abdominal surgery (Soh et al. 2024). Experts agree that an optimal prehabilitation program includes screening, assessment, intervention, and reassessment of multiple patient-related modifiable risk factors. Key screening components include physical fitness, nutritional status, psychological status, comorbidities (e.g., anemia, iron deficiency, glucose dysregulation, frailty), and substance use (e.g., smoking, alcohol). Patients with opportunities for optimization should undergo targeted assessment and intervention. The interventions are delivered as a care bundle, emphasizing the synergy between physical activity and nutrition (Driessens et al. 2024). Some research suggests that prehabilitation is most beneficial for high-risk patients (Berkel et al. 2018; Bongers et al. 2021; Barberan-Garcia et al. 2019). Although experts agree on the multimodal approach, there is no consensus on the content of a prehabilitation program and which patients should receive prehabilitation. Additionally, multidisciplinary involvement creates logistical and financial challenges, complicating large scale implementation.

The nationwide PROMISE-P study (ClinicalTrials.gov ID NCT05851534) aims to implement a nationwide standardized best-practice prehabilitation program across 13 Dutch hospitals performing pancreatic resections. Before this program can be integrated into routine care, it is crucial to evaluate existing prehabilitation practices. We therefore conducted a national survey among pancreatic surgeons as follows: (1) assess current preoperative screening practices, (2) explore surgeons’ opinions and knowledge of prehabilitation, and (3) identify existing prehabilitation programs for pancreatic surgery in the Netherlands. The findings will provide valuable insights into the current landscape of prehabilitation.

## Methods

### Study design

This descriptive cross-sectional study was conducted by the Department of Surgery of the Maastricht University Medical Centre (MUMC +)/Maastricht University (UM) and the University Medical Centre Groningen (UMCG) between June and December 2024. Since the study involved a survey studying current practices as perceived by health care professionals, ethical approval was not needed. Before starting the survey, we informed participants about its purpose, duration, data usage for publication, and data storage. This study was designed and reported in accordance with the Checklist for Reporting Results of Internet E-Surveys (Eysenbach 2004).

### Study setting and population

The survey was distributed to pancreatic surgeons at all 15 hospitals affiliated with the Dutch Pancreatic Cancer Group (DPCG) in the Netherlands. Participation was voluntary and strictly confidential. Only one pancreatic surgeon per hospital received the invitation to participate. This individual was selected based on their presumed expertise in pancreatic surgery and familiarity with their department’s prehabilitation practices and policies. We aimed to ensure that responses reflected institutional-level insights rather than individual opinions. We acknowledge that it may introduce a risk of selection bias, as the selected surgeon could present their hospital in a more favorable light. No incentives were provided for participation.

### Development of the survey

An online electronic survey (Additional file 1) was created via Qualtrics (Qualtrics, Provo, UT, USA). The survey content was developed by the authors, partially based on a previously used survey on prehabilitation practices for patients with colorectal cancer, developed by Molenaar et al. (2023). The survey was adapted to focus on pancreatic surgery, specifically the current daily practice of prehabilitation for patients undergoing pancreatic surgery. It included questions about general hospital-specific data, such as the number of pancreatic resections performed annually, the use of preoperative screening, surgeons’ knowledge and opinions on prehabilitation, and the design of prehabilitation programs in their hospital. Additional questions were asked if prehabilitation programs were already in use.

No personal data were collected. The survey consisted of 46 questions organized into 9 pages with 6 questions per page. The question types were either multiple-choice, checkbox, or open. The questions were not randomized; instead, logical ordering and adaptive questioning were used. The survey was tested thoroughly for usability and technical functionality by the study team, two surgeons from MUMC + , and two surgeons from UMCG. Revisions were made based on feedback. The complete survey can be found in Additional file 1.

### Data collection

The survey was distributed via email and completed online via Qualtrics (Qualtrics, Provo, UT, USA). Each question required an answer, but some questions were displayed conditionally based on previous responses. For example, if a surgeon answered “no” to whether any form of prehabilitation or preoperative screening was used in their hospital, no further questions were asked about the general and specific design of prehabilitation programs. To prevent multiple responses, each surgeon could respond only once to the invitation email, and the survey was not displayed again after submission. The data were pseudonymized before being exported from Qualtrics for analysis.

### Statistical analyses

The data are presented as numbers and percentages. Additional information given by the participating surgeons in open answer options was described. Since this is a descriptive study, only descriptive statistics were used for analysis. Data were analyzed using the Statistical Package for the Social Sciences (SPSS) for Windows (SPSS, V.28.0, IBM, SPSS, Chicago, IL, USA).

## Results

### Respondents

All 15 surgeons responded, resulting in a response rate of 100%. The survey took a median of 36 min to complete [range: 12–2187], with respondents able to save their progress, contributing to the variability in completion times. The participating hospitals perform a median of 65 surgeries per year [range: 45–100].

### Standard preoperative screening

In all hospitals, patients were screened for modifiable risk factors as part of standard preoperative care and thus not in the context of prehabilitation. The domains of the preoperative screening were physical fitness (93.3%), nutritional status (100%), anemia and iron deficiency (86.7%), frailty (80%), mental resilience (53.3%), glucose regulation (80%), smoking (86.7%), and alcohol use (86.7%). In the majority of the hospitals, an intervention was always applied when the screening outcome was abnormal and indicated an intervention (*n* = 9, 60%), whereas in 40% (*n* = 6) of the hospitals an intervention was not always applied even if the screening outcome for one or more domains was abnormal.

### Knowledge regarding prehabilitation

All of the responders were familiar with the term prehabilitation, and the majority (*n* = 11) agreed that prehabilitation is a structured, preoperative multimodal program for optimization. One responder stated that prehabilitation was “a mostly structured, preoperative program but with room for own decision-making.” Among the 11 respondents who answered that prehabilitation was “a structured, preoperative multimodal program for optimization,” the majority answered that the term multimodal means at least two interventions, followed by at least three interventions. Two recipients stated that multimodal means “structural screening of multiple domains” and “as many interventions as needed.” Only 33.3% knew the correct definition of a multimodal prehabilitation program as defined by Molenaar et al. (2023) (Molenaar et al. 2023) (see Table 1 for detailed information).

**Table 1:** Definition of prehabilitation according to all 15 respondents

	*N* (%)
Familiar with the term “prehabilitation”	15 (100)
Definition of prehabilitation	
Structured multimodal program	11 (73)
Any form of intervention	3 (20)
Other	1 (7)
Definition of multimodal^a^	
At least two interventions	5 (33)
At least three interventions	4 (27)

^a^This question was only asked to respondents who answered “a structural and multimodal program” to the question regarding the definition of prehabilitation, *n* = 11

### Current practice regarding prehabilitation

Twelve hospitals (80%) offered a prehabilitation program. Three hospitals did not offer a prehabilitation program. In two hospitals, this was due to financial and logistic reasons. One hospital stated that they were not yet convinced that prehabilitation was necessary for every patient. However, all three hospitals were willing to offer a prehabilitation program to their patients in the future.

**Fig. 1:**
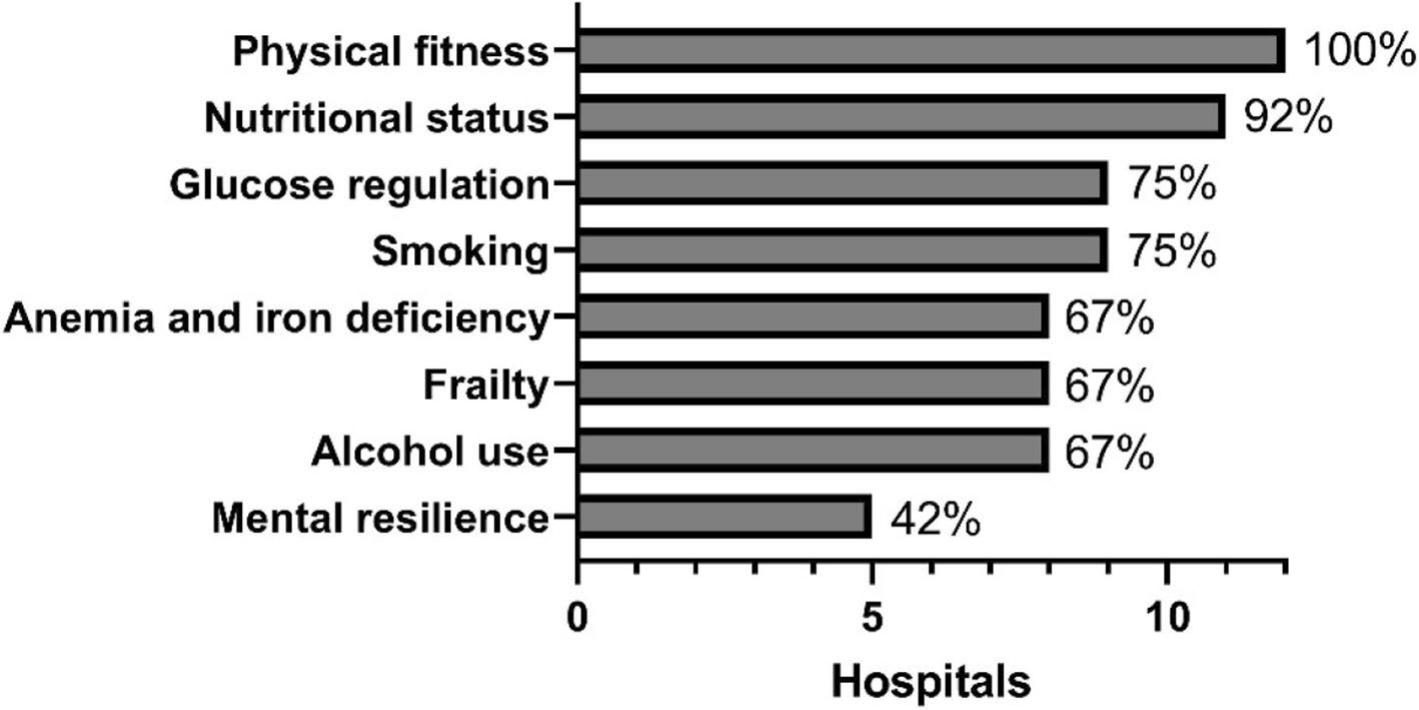
Included domains in prehabilitation programs

Prehabilitation programs were offered to all patients in the majority of hospitals (7/12 hospitals). Some hospitals offered prehabilitation programs only to subgroups. The subgroups included frail, older, high-risk patients and patients with comorbidities. A combination of patient subgroups was used in three cases (e.g., frailty combined with comorbidities). All prehabilitation programs included multiple domains, whereby physical fitness and nutrition were most often included and mental resilience was the least often included domain (Fig. 1).

The modality of the prehabilitation program was most frequently offered as a structured and individualized prehabilitation program followed by advice for the patient at home and a universal structured prehabilitation program. Delivery of prehabilitation was often performed in a combination of facilities, but was mostly delivered in health service facilities, followed by the hospital and, last, at home. More detailed information can be found in Table 2.

**Table 2:** Design and delivery of prehabilitation for 12 hospitals offering prehabilitation

	*n* (%)
Patient groups receiving prehabilitation	
All patients	7 (58)
Frail patients	3 (25)
Older patients	3 (25)
High-risk patients	4 (33)
Patients with comorbidities	3 (25)
Patients with perceived low physical fitness	1 (8)
Domains of prehabilitation	
Physical fitness	12 (100)
Nutritional status	11 (92)
Anemia and iron deficiency	8 (67)
Frailty	8 (67)
Mental resilience	5 (42)
Glucose regulation	9 (75)
Smoking	9 (75)
Alcohol use	8 (67)
Modality of prehabilitation	
Structured and individualized	7 (58)
Structured and universal	1 (8)
Advice for at home	4 (25)
Delivery of prehabilitation	
Health care facilities	9 (75)
Hospital	7 (58)
At home	6 (50)

### Prehabilitation screening and intervention

For each of the domains of prehabilitation, screening was used for physical fitness in 8 (67%) of the hospitals, for nutritional status in 11 (92%), anemia and iron deficiency in 8 (67%), frailty in 7 (58%), mental resilience in 4 (33%), glucose regulation in 8 (67%), smoking in 9 (75%), and alcohol use in 8 (67%) of the hospitals. For all of the domains, except for smoking and alcohol use where only one screening method was used, hospitals used two or more different screening methods. Three or more different interventions were used across hospitals. Only the intervention for frailty was uniform in all hospitals. Each hospital implemented a different prehabilitation program, differing in the included domains, screening methods, and interventions; no hospital had an identical prehabilitation program. More detailed information regarding all the different screening tools, assessments and interventions can be found in Additional file 2.

### Opinions regarding postponing surgery

Opinions regarding the willingness to postpone surgery to optimize the patient’s preoperative fitness differed across the respondents. For patients with malignant pancreatic and periampullary tumors, half of the respondents (53.3%) were willing to postpone surgery up to a maximum of 4 weeks, and the minority (20%) would extend this to a maximum of 6 weeks. A total of 26.7% of the respondents were willing to postpone surgery as long as necessary to optimize the patients’ preoperative overall fitness.

### Financing of prehabilitation

Most of the recipients agreed that prehabilitation should be financed by health insurance companies (87%), whereby one hospital stated that the patient should also finance a part of the prehabilitation program next to the health insurance company. Only one representative of a hospital stated that the patient should finance their entire prehabilitation program, and one representative of another hospital stated that the hospital should finance the prehabilitation program.

## Discussion

This nationwide survey among pancreatic surgeons investigated current preoperative prehabilitation practices before pancreatic surgery in the Netherlands. Our findings show that prehabilitation has been implemented in the majority of hospitals performing pancreatic surgery. All surgeons were familiar with the term prehabilitation, with the majority agreeing with the definition that “prehabilitation is a structured, preoperative multimodal program for optimization.” However, significant variability exists in the design and implementation of these programs across hospitals. The programs differ in terms of the included domains, screening methods, and applied interventions. This variation is notable given the standardized perioperative care in pancreatic surgery in the Netherlands, largely owing to the collaborative network of the DPCG. These findings suggest that prehabilitation is not yet established as a standard component of perioperative care. Furthermore, few hospitals have not implemented any prehabilitation program, underscoring the need for future efforts to integrate prehabilitation into routine clinical practice and thus preventing unwanted practice variation.

### Knowledge and opinions regarding prehabilitation

The findings of our study regarding knowledge of prehabilitation contrast with those of a survey among surgeons and anesthesiologists conducted in Japan (Sato et al. 2024). In their study, 38.7% of the surgeons were unaware of the term and content of prehabilitation, whereas in our study all surgeons were familiar with the term and content of prehabilitation. Our results regarding knowledge of prehabilitation are in line with those of a survey study among surgeons performing colorectal cancer surgery in the Netherlands, indicating that knowledge regarding prehabilitation might differ between countries and is high in the Netherlands (Molenaar et al. 2023).

Another important finding of this study was that surgeons are generally willing to delay surgery for pancreatic malignancies for prehabilitation. Our results regarding the willingness of surgeons to postpone surgery were comparable to those of other survey studies conducted among thoracic and colorectal surgeons (Molenaar et al. 2023; Shukla et al. 2020; Li et al. 2019). However, in practice, the average waiting time for surgery remains short, typically between 2 and 3 weeks, even in hospitals that have already implemented prehabilitation. To sufficiently optimize a patient’s condition before surgery, evidence suggests a prehabilitation program of a minimum of 3 weeks but preferably 4 weeks (Falz et al. 2022). This highlights a gap between opinions and clinical practice regarding delay of surgery. To address this, a paradigm shift in the preoperative phase is necessary, reframing the “waiting time” as “optimization time.”

### Variation in prehabilitation programs

Our data revealed variability in the design and content of the implemented prehabilitation programs in the Netherlands. This corresponds with previous studies on prehabilitation before pancreatic surgery (Deprato et al. 2022; Bundred et al. 2020). The domains that were most often included in prehabilitation programs in the Netherlands were physical fitness and nutrition. To illustrate the differences in content, five different screening methods for physical fitness and three different screening methods for nutritional status were used. The screening methods for physical fitness were mostly based on patient characteristics or subjective assessments and not on validated questionnaires or tests. For both domains, three different interventions were used. These results indicate a lack of uniformity in prehabilitation programs. For physical fitness, the current national guideline on “Physical fitness of patients with and after cancer” states that screening should be based on validated questionnaires such as the Duke Activity Status Index (DASI), which none of the hospitals in our study used (Fysieke fitheid van mensen met en na kanker 2025). The current guidelines on “pancreatic cancer” in the Netherlands state that screening for malnutrition should be performed with the Patient-Generated Subjective Global Assessment (PG-SGA) short form, which was used by only 25% of the hospitals in our study (Vereniging and voor Heelkunde (NVvH), 2019). Our findings indicate that despite the availability of national guidelines, these guidelines are not implemented. This highlights the need for the development of a uniform standardized prehabilitation program and supervision of actual implementation.

An additional important observation is that psychological support to enhance mental resilience is not routinely included, consistent with preliminary findings from a systematic review on psychological interventions in prehabilitation (Hirst et al. 2024). Yet, psychological factors such as anxiety and depression negatively impact postoperative outcomes (Levett and Grimmett 2019). A growing body of evidence supports psychological prehabilitation in improving these postoperative outcomes (Hall et al. 2025). Therefore, the inclusion of the psychological domain, supported by a psychologist as part of the multidisciplinary prehabilitation team, should be considered essential.

### Challenges of implementation

Several challenges hinder the implementation of prehabilitation as part of standard preoperative care, mainly due to financial and logistical constraints. Currently, prehabilitation is not covered by health insurance in the Netherlands, particularly in the domain of physical fitness. Patients are not reimbursed for preoperative exercise training provided by physiotherapists, which is a critical component of prehabilitation. While internal hospital referrals for other domains are covered by health insurance, the lack of financial support for supervised exercise training creates inequities in care and ultimately health outcomes. The logistical constraints that challenge implementation include limited access to resources (equipment, qualified healthcare workers), compliance barriers, and coordination challenges. The PACAP-1 study revealed that adherence to the best practices after implementation is challenging at the national scale (Mackay et al. 2024). Beyond clinical trials, implementation studies have identified important facilitators and barriers to the successful integration of prehabilitation (Sontag et al. 2024; Fuchs et al. 2024). Understanding these context-specific factors is essential for developing sustainable programs. Tools like the BARRIERS framework can help to tailor implementation strategies to real-world conditions and bridge the gap between evidence and practice (Funk et al. 1991).

Next to addressing barriers, to improve the implementation of and adherence to prehabilitation programs, implementation frameworks and structured quality assurance processes should be developed (Mapping and identifying quality and inequality in prehabilitation for cancer surgery: evidence for improvement. 2023). These could include strategies such as embedding prehabilitation into existing care pathways, defining performance indicators, and leveraging hospital accreditation or national audit cycles to ensure consistent delivery and adherence (Bates et al. 2020).

### Strengths and limitations

This study has several strengths. First, all hospitals in the Netherlands performing pancreatic surgery were included, ensuring comprehensive nationwide coverage and minimizing selection bias at the institutional level. Second, the survey provided detailed insights into the opinions, beliefs, and current practices of pancreatic surgeons regarding prehabilitation. Importantly, responses were collected from surgeons identified as the most knowledgeable about prehabilitation at their respective institutions, enhancing the reliability and relevance of the data. However, selection bias may have occurred, as only by these authors chosen surgeons with a particular interest in or awareness of prehabilitation were invited to respond to the survey, possibly skewing the results toward more favorable perspectives on prehabilitation. Despite its strengths, the study has several limitations. The use of open-ended survey questions posed challenges in organizing and analyzing responses, as the answers were often imprecise or lacked sufficient detail. Additionally, the small sample size of 15 respondents limits the generalizability of the findings.

### Future directions

A recent systematic review on prehabilitation before pancreatic surgery concluded that there is a need for standardization of prehabilitation in terms of program content and measured outcomes (Refaat et al. 2024). Standardization would allow comparisons across studies, both nationally and internationally. Despite promising findings, the evidence supporting prehabilitation remains limited, and further research, preferably large uniform trials, is needed.

The ongoing PROMISE-P study can be an important step forward. This large-scale, nationwide study aims to implement a standardized prehabilitation program and measure outcomes across the Netherlands. The PROMISE-P study will provide valuable insights into the effectiveness of prehabilitation in improving postoperative outcomes in patients undergoing pancreatic surgery. These insights hopefully will also result in reimbursing prehabilitation programs in the future. It is important to realize that the study outcomes are linked to the full and correct implementation of the protocol and all its different domains. Furthermore, the survey findings indicate that prehabilitation is already a recognized and implemented concept in many hospitals across the Netherlands. This could be a potential challenge for the PROMISE-P study, as several participating hospitals already offer some form of prehabilitation during the control period. As a result, the anticipated impact of prehabilitation on outcomes such as functional recovery and hospital stay duration may be lower than initially expected during the design of the PROMISE-P study. It also shows that the ideal study in which prehabilitation is studied with a control group in which no prehabilitation is conducted cannot be executed in the current practice; the design of the PROMISE-P study is then the best possible approach. The survey revealed significant variations in content of prehabilitation programs, indicating that hospitals currently offer suboptimal prehabilitation programs that do not address all domains. The PROMISE-P study standardizes prehabilitation, creating an optimized program and potentially improving compliance through supervision from the local team supported by the study team.

## Conclusion

In conclusion, our survey results suggest that pancreatic surgeons have knowledge of prehabilitation, but high variability exists in current practice regarding prehabilitation programs. Prehabilitation differed in terms of the included domains, screening methods, and applied interventions. There is a need for a uniform standardized prehabilitation program to implement prehabilitation in the standard preoperative care pathway and enable comparisons of results across hospitals. The ongoing PROMISE-P trial will provide valuable insights.

## Supplementary Information

Additional file 1. Questionnaire regarding prehabilitation

Additional file 2. Detailed information regarding screening, assessment and interventions. Table 1. Details regarding screening, assessment and intervention of each domain used by the hospitals providing prehabilitation (*n*=12).

## Data Availability

The datasets used and/or analyzed during the current study are available from the corresponding author on reasonable request.

## Abbreviations

MUMC +: Maastricht University Medical Centre
UMCG: University Medical Centre Groningen
UM: Maastricht University
ERAS: Enhanced recovery after surgery
PROMISE-P: Preoperative optimization of modifiable risk factors in surgery of the pancreas: the implementation of best practice before pancreatic resection
DPCG: Dutch Pancreatic Cancer Group
SPSS: Statistical Package for the Social Sciences
PG-SGA: Patient-Generated Subjective Global Assessment
DASI: Duke Activity Status Index
PACAP-1: Dutch Pancreatic Cancer Project
