# Supplementary material for: Prehabilitation before pancreatic surgery in the Netherlands: insights from a nationwide survey among pancreatic surgeons
Source: Perioper Med (Lond). 2025 Jul 31;14:82. doi: 10.1186/s13741-025-00569-x (PMC12312366; doi:10.1186/s13741-025-00569-x)
Supplement: Supplementary file 1 — Additional file 1. Questionnaire regarding prehabilitation [file 13741_2025_569_MOESM1_ESM.docx]

**Additional file 1. Questionnaire regarding prehabilitation**

**Introduction**

Thank you for taking the time to complete this questionnaire! Through this questionnaire, we would like to inventory what the current standard of care is in your hospital regarding prehabilitation or preoperative optimization for patients prior to pancreatic surgery. Completing the questionnaire will take approximately 5 to 10 minutes depending on the answers given. By completing this questionnaire, you agree to the use of anonymized data for scientific research.

1. What is the name of your pancreatic center?
   *The purpose of this question is to document the answers. This information will be treated strictly confidentially and not used for publication.* *Select one answer.*
   - Amphia Hospital
   - Amsterdam UMC
   - Catharina Hospital
   - Erasmus MC
   - Isala Clinics
   - Jeroen Bosch Hospital
   - Leiden UMC
   - Maasstad Hospital
   - Maastricht UMC
   - Medical Spectrum Twente
   - OLVG
   - Radboud UMC
   - RAKU (UMCU & St. Antonius)
   - UMC Groningen
   - MCL
2. How many pancreatic surgeries does your hospital perform per year (all pancreatic resections for both benign and malignant conditions)? *Enter a number.*

**Preoperative optimization in your hospital**

The following section concerns the preoperative examination of patients undergoing pancreatic resection in your hospital. Specifically, it is about preoperative screening and intervention(s) as part of standard care.

1. Are patients prior to pancreatic surgery in your hospital screened for the following domains as part of standard care? *Select one or more answers.*
   - Physical fitness
   - Nutritional status
   - Anemia and iron deficiency
   - Frailty
   - Mental resilience
   - Glucose regulation
   - Alcohol use
   - Smoking
   - Other, namely...
2. Is an intervention applied if the screening of a domain is abnormal?
   *Examples of domains include, but are not limited to: nutritional status, frailty, physical fitness, mental resilience, glucose regulation, intoxications.* *Select one or more answers.*
   - Yes (an intervention is always applied when needed for one or more domains)
   - Sometimes (an intervention is not always applied even if the screening for one or more domains is abnormal)
   - No (no intervention is applied)

**Prehabilitation**

1. Are you familiar with the term 'prehabilitation'? *Select one answer.*
   - Yes
   - No
2. What do you mean by 'prehabilitation'? *Select one answer. (only shown if answer to question 5 is ‘Yes’)*
   - Any form of preoperative interventions for optimization
   - A structured, preoperative multimodal program for optimization
   - Other, namely...
3. What do you mean by multimodal? *Select one answer. (only shown if answer to question 6 is ‘A structured, preoperative multimodal program for optimization’)*
   - At least two interventions
   - At least three interventions
   - Other, namely...
4. Is a prehabilitation/preoperative optimization program offered to (some of) the patients prior to pancreatic surgery in your hospital? *Select one answer.*
   - Yes
   - No

**Prehabilitation in your hospital (questions 9 through 13 are only shown if answer to question 8 is ‘Yes’)**

The following questions are about prehabilitation for patients with pancreatic tumors in your hospital.

1. Which (sub)groups are eligible for prehabilitation? *Select one or more answers.*
   - All patients
   - Frail patients
   - Older patients
   - High-risk patients
   - Patients with comorbidities
   - Other, namely...
2. How do you define this subgroup (for example, age > 70 years, ASA > 3, etc)? *Open question.*
3. Which domains are included in the prehabilitation program? *Select one or more answers.*
   - Physical fitness
   - Nutritional status
   - Anemia and iron deficiency
   - Frailty
   - Mental resilience
   - Glucose regulation
   - Alcohol use
   - Smoking
   - Other, namely...
4. What is the design of the prehabilitation interventions? *Select one or more answers.*
   - Advice for the home setting
   - A structured and standard program, the same for all patients
   - A structured and individualized program, tailored for each patient
   - Other, namely...
5. In what setting is the program offered? *Select one or more answers.*
   - In the hospital
   - In primary care
   - In the gym
   - Home setting

**Prehabilitation in your hospital (questions 14 through 21 are only shown if answer to question 8 is ‘No’)**

The following questions are about not (yet) offering prehabilitation for patients with pancreatic tumors in your hospital.

1. What is the reason you do not offer a prehabilitation program to patients with pancreatic tumors in your hospital? *Select one answer.*
   - We haven't thought about it before
   - For logistical reasons
   - For financial reasons
   - Other, namely...
2. Would you like to offer a prehabilitation program to patients with pancreatic tumors? *Select one answer.*
   - Yes
   - No
3. What is the reason you do not want to offer a prehabilitation program in your hospital? *Select one or more answers. (only shown if answer to question 15 is ‘No’)*
   - For logistical reasons
   - For financial reasons
   - I cannot convince my colleagues of the benefit of prehabilitation
   - I am not convinced of the benefit of prehabilitation
   - Other, namely...
4. Which (sub)groups should be eligible for prehabilitation? *Select one or more answers. (only shown if answer to question 15 is ‘Yes’)*
   - All patients
   - Frail patients
   - Older patients
   - High-risk patients
   - Patients with comorbidities
   - Other, namely...
5. Would you triage patients before starting a prehabilitation program? *Select one answer. (only shown if answer to question 15 is ‘Yes’)*
   - Yes
   - No
6. Which domains should be included in the prehabilitation program? *Select one or more answers. (only shown if answer to question 15 is ‘Yes’)*
   - Physical fitness
   - Nutritional status
   - Anemia and iron deficiency
   - Frailty
   - Mental resilience
   - Glucose regulation
   - Alcohol use
   - Smoking
   - Other, namely...
7. What should be the design of the interventions? *Select one or more answers. (only shown if answer to question 15 is ‘Yes’)*
   - Advice for the home setting
   - A structured and standard program, the same for all patients
   - A structured and individualized program, tailored for each patient
   - Other, namely...
8. In what setting should the program be offered? *Select one or more answers. (only shown if answer to question 15 is ‘Yes’)*
   - In the hospital
   - In primary care
   - In the gym
   - Home setting

**Financial and logistical questions**

1. How should prehabilitation be financed? *Select one answer.*
   - Patients pay for their own prehabilitation program
   - The insurer covers the costs
   - The hospital pays for the prehabilitation program
   - Other, namely...
2. Would you postpone surgery for malignant pancreatic and periampullary tumors to optimize the patient's preoperative condition? *Select one answer.*
   - Yes, up to 2 weeks from diagnosis
   - Yes, up to 4 weeks from diagnosis
   - Yes, up to 6 weeks from diagnosis
   - Yes, up to 8 weeks from diagnosis
   - Yes, as long as necessary
   - No, I would not postpone the operation

**Prehabilitation - In depth questions**

With the following questions, we would like to inventory what the prehabilitation program in your hospital looks like. In prehabilitation, the principle of screening for modifiable risk factors is often used, followed by a follow-up examination to better map this risk factor, followed by an intervention to improve this risk factor. We would like to know if screening, a follow-up examination, and an intervention take place in your hospital, and what this entails.

**Prehabilitation - Physical Fitness (question 24 through 27 are only shown if ‘Physical fitness’ is checked on at question 11)**

1. Which factors do you consider in your decision to further examine patients for physical fitness? *Select one or more answers.*
   - All patients receive follow-up examination
   - Age
   - Co-morbidity
   - Trajectory of preoperative chemotherapy and/or radiotherapy
   - History of physical activity
   - Body composition
   - Other, namely...
2. Which follow-up examination(s) do you use to determine physical fitness? *Select one or more answers.*
   - Stair climbing test
   - Time up and go
   - 6-minute walk test
   - Steep ramp test
   - Cardiopulmonary exercise test (CPET)
   - Questionnaire, note under 'Other' which one you use
   - No follow-up examination takes place
   - Other, namely...
3. Explain what cut-off values you use in screening and follow-up examination of physical fitness? (for example, age above 70 years and CPET VO2peak below 18 ml/kg/min). *Open question.*
4. What intervention(s) do you apply to improve the patient's physical fitness? *Select one or more answers.*
   - These patients are no longer operated on and referred for palliative therapy
   - These patients are referred to a tertiary referral center for further treatment due to increased perioperative risk
   - These patients are encouraged to move a lot at home independently
   - Referral to a physiotherapist chosen by the patient for training
   - Referral to a physiotherapist chosen by the hospital who has experience with preoperative optimization
   - No intervention takes place
   - Other, namely...

**Prehabilitation – Nutrition (question 28 through 31 are only shown if ‘Nutritional status’ is checked on at question 11)**

1. How do you screen patients for a reduced nutritional status? *Select one or more answers.*
   - This is not screened for
   - No screening for weight loss
   - No screening for pancreatic carcinoma
   - Short Nutritional Assessment Questionnaire (SNAQ)
   - Malnutrition Universal Screening Tool (MUST)
   - Patient-Generated Subjective Global Assessment (PG-SGA) Short Form
   - Other, namely...
2. What follow-up examination do you use to determine nutritional status? *Select one answer.*
   - Nutritional assessment by the dietitian
   - No follow-up examination takes place
   - Other, namely...
3. Explain what cut-off values you use in screening for nutritional status (for example, MUST > 4). *Open question.*
4. What intervention(s) do you apply to improve the patient's nutritional status? *Select one or more answers.*
   - Referral to dietitian in the hospital
   - Referral to dietitian in primary care
   - Nutritional advice
   - No intervention takes place
   - Other, namely...

**Prehabilitation - Mental Resilience (question 32 through 34 are only shown if ‘Mental resilience’ is checked on at question 11)**

1. How do you screen patients for low mental resilience? *Select one or more answers.*
   - This is not screened for
   - With the hospital anxiety and depression scale (HADS)
   - With the State-Trait Anxiety Inventory (STAI)
   - Other, namely...
2. Explain what cut-off values you use in screening for low mental resilience (for example: HADS > 8). *Open question.*
3. What intervention(s) do you apply to improve the patient's mental resilience? *Select one or more answers.*
   - Advice and conversation with nurse/nurse specialist/case manager
   - Referral to medical psychologist in the hospital
   - Referral to general practitioner/POH GGZ
   - E-learning about mental resilience
   - No intervention takes place
   - Other, namely...

**Prehabilitation - Anemia and Iron Deficiency (question 35 through 38 are only shown if ‘Anemia and iron deficiency’ is checked on at question 11)**

1. How do you screen patients for (iron deficiency) anemia? *Select one answer.*
   - Hb
   - Hb and iron status
   - Hb, iron status, vitamin B12, and folic acid
   - This is not screened for
   - Other, namely...
2. Explain what cut-off values you use in screening for (iron deficiency) anemia. *Open question.*
3. What intervention(s) do you apply for (iron deficiency) anemia? *Select one or more answers.*
   - Oral iron supplementation
   - I.V. iron supplementation
   - Supplementation of red blood cells shortly before or during surgery
   - Treatment by the general practitioner
   - Treatment by the internist/hematologist
   - No intervention takes place
   - Other, namely...
4. When does the patient receive iron supplementation? *Select one answer.*
   - If both iron and Hb are abnormal
   - Only if iron is abnormal
   - Only if Hb is abnormal

**Prehabilitation – Frailty (question 39 through 41 are only shown if ‘Frailty’ is checked on at question 11)**

1. How do you screen patients for frailty? *Select one or more answers.*
   - This is not screened for
   - Age
   - Age and presence of comorbidity(ies)
   - Groningen Frailty Indicator
   - Geriatric 8
   - Robinson's Frailty Score
   - Clinical Frailty Scale
   - Other, namely...
2. Explain what cut-off values you use in screening for frailty. *Open question.*
3. What intervention(s) do you apply for frailty? *Select one or more answers.*
   - Referral to a geriatrician/specialist in elderly medicine
   - Referral to the general practitioner
   - No intervention takes place
   - Other, namely...

**Prehabilitation – Intoxications**

1. What intervention(s) do you apply for patients who smoke? *Select one or more answers. (only shown if ‘smoking’ is checked on at question 11)*
   - Advice at the outpatient clinic to stop smoking
   - Referral to a lifestyle desk
   - Referral to a 'stop smoking' clinic
   - Referral to the general practitioner
   - Referral to MediPro, Sinefuma, or other organization
   - No intervention takes place
   - Other, namely...
2. What intervention(s) do you apply for patients who drink alcohol? *Select one or more answers. (only shown if ‘alcohol’ is checked on at question 11)*
   - Advice at the outpatient clinic to stop drinking alcohol
   - Referral to a lifestyle desk
   - Referral to the general practitioner
   - Referral to an addiction specialist
   - No intervention takes place
   - Other, namely...

**Prehabilitation - Glucose Regulation (question 44 through 46 are only shown if ‘Glucose regulation’ is checked on at question 11)**

1. How do you screen patients for impaired glucose regulation? *Select one or more answers.*
   - Fasting blood glucose value
   - HbA1c
   - No screening for patients known with diabetes mellitus, as all these patients are referred
   - No screening for patients receiving pancreatic resection, as all these patients are referred
   - No screening takes place
   - Other, namely...
2. Explain what cut-off values you use in screening for impaired glucose regulation. *Open answer.*
3. What intervention(s) do you apply for patients with impaired glucose regulation? *Select one or more answers.*
   - Diet and lifestyle advice at the outpatient clinic
   - Referral to a diabetes nurse in the hospital
   - Referral to an internist/endocrinologist
   - Referral to the general practitioner
   - No intervention takes place
   - Other, namely...
