# Supplementary material for: Prehabilitation before pancreatic surgery in the Netherlands: insights from a nationwide survey among pancreatic surgeons
Source: Perioper Med (Lond). 2025 Jul 31;14:82. doi: 10.1186/s13741-025-00569-x (PMC12312366; doi:10.1186/s13741-025-00569-x)
Supplement: Supplementary file 2 — Additional file 2. Detailed information regarding screening, assessment and interventions. Table 1. Details regarding screening, assessment and intervention of each domain used by the hospitals providing prehabilitation (n=12). [file 13741_2025_569_MOESM2_ESM.docx]

Additional file 2. Detailed information regarding screening, assessment and interventions

*Table 1. Details regarding screening, assessment and intervention of each domain used by the hospitals providing prehabilitation (n=12).*

|  | **Screening performed** | **Screening based on** | **Assessment tools** | **Interventions** |
| --- | --- | --- | --- | --- |
| **Physical fitness** | 8 (67) | Age, 7 (58)  Comorbidities, 8 (67)  Neoadjuvant chemo(radio)therapy, 6 (50)  Level of physical activity, 7 (58)  Body composition, 1 (8) | SRT, 4 (33)  CPET, 1 (8)  Stair climb test, 2 (16)  TUG, 1 (8)  6-MWT, 1 (8)  Grip strength, 1 (8)  1 RPM, 1 (8) | Training advice, 8 (67)  Referral to physician of patient’s choice, 4 (33)  Referral to physician of caregiver’s choice, 7 (58) |
| **Nutritional status** | 11 (92) | SNAQ, 7 (58)  MUST, 3 (25)  PG-SGA SF, 3 (25) | Nutritional assessment by dietician, 11 (92) | Referral to dietician in hospital, 10 (83)  Referral to dietician to health care facility, 3 (25)  Dietary advice, 4 (33) |
| **Anemia and iron deficiency** | 8 (67) | Hemoglobin, 1 (8)  Hemoglobin and iron status, 5 (42)  Hemoglobin, iron status, vitamin B12 and folic acid, 2 (17) | NA | Oral iron supplementation, 3 (25)  Intravenous iron supplementation, 7 (58)  RBC supplementation, 2 (17) |
| **Frailty** | 7 (58) | Age, 3 (25)  Age and comorbidities, 2 (17)  GFI, 2 (17)  RFS, 1 (8)  G8, 4 (33)  6CIT, 1 (8) | NA | Referral to geriatrician, 7 (58) |
| **Mental resilience** | 4 (33) | HADS, 3 (25)  History taking, 1 (8) | NA | Consultation with nurse practitioner, 3 (25)  Referral to psychologist, 2 (17)  Referral to GP, 1 (8) |
| **Glucose regulation** | 8 (67) | Fasting glucose, 6 (50)  HbA1c, 4 (33) | NA | Dietary and lifestyle advice, 1 (8)  Referral to nurse practitioner DM, 6 (50)  Referral to internal medicine, 5 (42)  Referral to GP, 1 (8) |
| **Smoking** | 9 (75) | History taking, 9 (75) | NA | Advice to quit, 5 (42)  Referral to lifestyle clinic, 3 (25)  Referral to ‘quit smoking’ outpatient clinic, 3 (25)  Referral to GP, 3 (25)  Referral to external organization, 6 (50) |
| **Alcohol use** | 8 (67) | History taking, 8 (67) | NA | Advice to quit, 7 (58)  Referral to lifestyle clinic, 2 (17)  Referral to GP, 1 (8) |

Values are depicted as numbers (%).

6CIT = 6-Item Cognitive Impairment Test, 6-MWT = 6-Minute Walk Test, CPET = Cardiopulmonary Exercise Test, G8 = Geriatric 8, GFI = Groningen Frailty Indicator, HADS = Hospital Anxiety and Depression Scale, HbA1c = Glycated Hemoglobin, MUST = Malnutrition Universal Screening Tool, PG-SGA SF = Patient-Generated Subjective Global Assessment Short Form, RBC = Red Blood Cell, RFS = Robinson Frailty Score, RPM = Repetition Maximum, SNAQ = Short Nutritional Assessment Questionnaire, SRT = Steep Ramp Test, TUG = Timed Up and Go Test.
